# Drug-related problem characterization and the solved status associated factor analysis in a pharmacist-managed anticoagulation clinic

**DOI:** 10.1371/journal.pone.0270263

**Published:** 2022-08-15

**Authors:** Ju-Chieh Wung, Hsin-Chung Lin, Chia-Chen Hsu, Chia-Chieh Lin, Szu-Yu Wang, Shih-Lin Chang, Yuh-Lih Chang

**Affiliations:** 1 Department of Pharmacy, Taipei Veterans General Hospital, Taipei, Taiwan; 2 School of Pharmacy, National Defense Medical Center, Taipei, Taiwan; 3 Department of Pharmacy, National Yang Ming Chiao Tung University, Taipei, Taiwan; 4 Department of Medicine, Heart Rhythm Center and Division of Cardiology, Taipei Veterans General Hospital, Taipei, Taiwan; 5 School of Medicine, National Yang Ming Chiao Tung University, Taipei, Taiwan; 6 Institute of Pharmacology, College of Medicine, National Yang Ming Chiao Tung University, Taipei, Taiwan; Leiden University Medical Center, NETHERLANDS

## Abstract

Drug-related problems (DRPs) in a pharmacist-managed anticoagulation clinic (AC) have not been extensively studied. We aimed to characterize the DRPs in a pharmacist-managed AC, identify the factors associated with the solved status of DRPs, and analyze the secondary outcomes, including the safety and efficacy of AC service. The patients receiving services at a pharmacist-managed AC in a medical center for the first time from March 2019 to August 2020 were reviewed retrospectively. The DRPs were retrieved from a self-developed Intelligent AC Service System and classified according to the Pharmaceutical Care Network Europe Foundation v9.0 classification system. Logistic regression models were performed to identify the potential factors associated with the solved status of DRPs. A total of 78 direct oral anticoagulant (DOAC) and 34 warfarin users were included. The major types of DRPs identified at the initial service were adverse drug events (ADEs) (68.4%) and untreated symptoms or indications (14.8%) in the DOAC group, and ADEs (51.6%) and suboptimal effect of drug treatment (38.7%) in the warfarin group. The rates of totally solved DRPs were 56.8% and 51.6% in the DOAC and warfarin groups, respectively. According to the multivariable analysis, receiving AC services 3 times or more in 180 days (OR 3.11, 95% CI 1.30–7.44) was associated with the totally solved status of DRPs in the DOAC group, but no relevant factor was identified in the warfarin group. The secondary outcomes showed that DOAC users demonstrated fewer thromboembolism events, major bleeding, and bleeding-related hospitalizations after AC services, whereas the warfarin users increased percentage time in therapeutic range (TTR% 55.0% vs. 74.6%, P = 0.006) after AC services. These findings may be utilized to develop DOAC and warfarin AC services.

## Introduction

Warfarin has been the most commonly used oral anticoagulant since its introduction in 1954. Due to its narrow therapeutic index, complex pharmacokinetic/dynamic profile, and difficulty in managing warfarin-associated problems, pharmacist-managed anticoagulation clinics have been established and implemented worldwide to improve anticoagulation control. Since direct oral anticoagulants (DOACs) were available in 2010, their effectiveness and safety have been demonstrated to be superior or non-inferior to warfarin in pivotal studies [1–4]; thus, DOACs are recommended over warfarin for stroke prevention in patients with in atrial fibrillation (AF), except for those with mechanical heart valves or moderate-to-severe mitral stenosis [5]. DOACs have characteristics of fixed dosing regimen, fewer drug-food interactions, and a predictable pharmacokinetic profile with no routine coagulation tests in clinical practice. Therefore, the choice of DOACs has the net benefit compared to warfarin [6]. Recent studies show the population of DOAC users is growing, indicating the preference for DOACs in patients with newly diagnosed AF [7–9]. The increasing number of DOAC users increases the need for pharmacy services by anticoagulation clinics (ACs).

On the other hand, pharmacist-led medication review of drug-related problems (DRPs) has become a key strategy for preventing and reducing harm [10]. A DRP is a drug therapy-related event or circumstance that interferes or may interfere with desired health outcomes. While recent studies of DOAC pharmacy service mainly focus on medication adherence and dosage appropriateness [11–13], only a few assessed drug therapy problems and related resolutions [14]. Thus, a thorough characterization of DRPs and the follow-up of corresponding interventions and solved status may provide crucial information for improving pharmacy services in AC.

Here, we aimed to assess the DRPs of patients who received initial AC services and analyze the corresponding interventions and problem solved status within 180 days after the initial services. Then, we aimed to identify the factors associated with the solved status of DRPs. From our findings we gained knowledge on the frequent types of DRPs and their common causes, which can be utilized to improve AC services.

## Methods

### Ethics statement

This study was approved by the Institutional Review Board of Taipei Veterans General Hospital (TPEVGH IRB No. 2020-08-010AC). Since the research posed no more than minimal risk to the participants and did not involve medical procedures, the review board agreed to waive the written informed consent from the patients.

### Study design

This retrospective study was conducted at Taipei Veterans General Hospital in Taiwan, a tertiary medical center. The patients receiving pharmacist-managed AC services for the first time from March 2019 to August 2020 were recruited. Each participant’s medical records from 180 days before to 180 days after the initial service to AC service were collected.

### The anticoagulation clinic in Taipei Veterans General Hospital

The pharmacist-managed AC at Taipei Veterans General Hospital (TPEVGH), established in 2012, provides DOAC and warfarin services to the patients referred by physicians from cardiovascular and cardiovascular surgery divisions in an ambulatory setting. The referral criteria included initial use of oral anticoagulant, adverse drug event management, improvement in medication adherence, drug interaction management, oral anticoagulant (OAC) interruption before invasive procedures, lifestyle management, OAC follow-up, and OAC counseling (S1 Fig). All physicians were required to agree to the DOAC dosing criteria in the package inserts before referring patients.

The initial service contains three domains, patient information collection, evaluation, and intervention. Patient information collection involved obtaining a patient’s medical history, including comorbidities, thromboembolism events, major bleeding events, bleeding-related hospitalizations, history and indication of OACs, current drug profile, lifestyle, vital signs, and lab data. All patient information was collected by retrieving medical histories from the Hospital Information System at TPEVGH or National Health Insurance database in Taiwan using the patients’ health ID cards. The pharmacists would confirm the information with the patients. Lastly, the lifestyle information was self-reported by the patients. The evaluation domain involved drug-related problem assessment, CHA_2_DS_2_-VASc and HAS-BLED scoring, percentage time in therapeutic range (TTR%), and international normalized ratio (INR) test ratio within 180 days before the first service.

The follow-up service had the same service structure as the initial service for collecting patient information between services and evaluating the status of the identified DRP. The initial service was inperson only, and the follow-up service was inperson or over the phone. All AC services were documented in the SOAP format and uploaded to the health information system in TPEVGH. All the information and data in each service were imported to the self-developed Intelligent Anticoagulation Clinic (AC) Service System (Patent No. M574731).

### Date collection and definitions

Patient information and data were retrieved from the self-developed Intelligent AC Service System, Electronic Medical Records, and the Hospital Information System at TPEVGH. The DRPs were detected and characterized according to the criteria of the Pharmaceutical Care Network Europe working group on drug-related problems (PCNE-DRP) v9.0 classification system [15].

PCNE-DRP v.9.0 includes 3 primary domains for problems, 9 primary domains for causes, 5 primary domains for interventions, 3 primary domains for acceptance of the intervention proposals, and 4 primary domains for the status of a DRP. In addition, there are 7 grouped subdomains for problems, 43 grouped subdomains for causes, 17 grouped subdomains for interventions, 10 subdomains for intervention acceptance, and 7 subdomains for DRP status. Each patient may have multiple DRPs. Each DRP may have multiple causes that lead to multiple interventions but only one outcome status. Lastly, each intervention has an acceptance status.

All the patient information and data at the initial service were reviewed to identify DRPs and their causes. Regarding the classification of interventions, documentation of communication with physicians was classified as an intervention at the prescriber level. Meanwhile, documentation of patient education or consultation for the corresponding DRP was classified as an intervention at the patient level. In addition, documentation of suggestions of prescription adjustment was classified as an intervention at the drug level.

Then, we followed the DRP status for 180 days after the initial service by reviewing each follow-up service documentation in the Intelligent AC Service System, Electronic Medical Records, and the Hospital Information System at TPEVGH. An accepted intervention was classified as no refusal or decline records to the intervention. A fully implemented intervention at each level was classified as the treatments, patient responses, or prescription adjustments following the corresponding intervention. An unimplemented intervention was classified as no actions related to the intervention. If a relevant action was between fully implemented and not implemented, it was classified as partially implemented. If a patient was lost to the follow-up or canceled/postponed invasive procedures until after the follow-up period, the implementation was classified as unknown. Finally, we classified the status of DRPs by reviewing the follow-up service, which was closest to the 180 days after the initial service. If a patient was lost to the follow-up or canceled/postponed invasive procedures until after the follow-up period, the status of the corresponding DRP was classified as not known. For the patients using warfarin, a recent within-range INR value was the prerequisite condition to a defined totally solved status. A DRP, in problem domain P1.2, P1.3, or P3.2, was classified as having the totally solved status under appropriate prescription adjustment. A DRP in problem domain P2.1 was classified as totally solved status when the adverse drug event was properly controlled or the risk or cause of ADE was completely removed. Otherwise, the solved status of DRP was classified as partially solved. According to our service protocol, a phone follow-up was initiated if the patient did not return to our hospital as scheduled. In addition, the survey of studied clinical events was performed during the follow-up service by phone, and the survey results were documented in the standardized SOAP format. All DRPs were identified by two authors, who were JC Wung and HC Lin, independently. Any discrepancy DRP classification or outcomes were discussed to reach an agreement between the two reviewers or consulted with a third person on the research team to reach a final decision.

The thromboembolism events, major bleeding events, and bleeding-related hospitalizations of a patient within 180 days before the first service were collected at the first service. The events within 180 days after the first service were collected at each followed-up service using the same approach. In addition, we calculated TTR% for warfarin users 180 days before and after the first service.

### Outcomes

The primary outcomes included DRP classification, the status of DRPs, and the factors associated with the solved status of DRPs. On the other hand, the secondary outcomes are the safety and efficacy of anticoagulation pharmacy services, including a comparison of thromboembolism events, major bleeding events, and bleeding-related hospitalizations within 180 days before and after the first AC visit. The warfarin service also included a comparison of TTR% within 180 days before and after the first AC service. Among patients taking warfarin, the improvement of their TTR to more than 70% are associated with reduced bleeding risk and increased efficacy [16]. In a rivaroxaban clinical trial, the average TTR on the Asian population is 52% [17]. Thus, we used 2 TTR cut-offs, 50% and 70%, to analyze the improvement in TTR control.

### Statistical analysis

All data were extracted and collected using Microsoft Office Excel 2013 (Microsoft, Redmond, WA, USA). The data were further processed and analyzed using the SAS^®^ 9.4 (SAS Institute Inc, Cary, NC, USA). Since the most used indications between the warfarin and DOAC groups were different, the innate patient characteristics, service, and analyses were displayed separately. Descriptive statistics was used to summarize the characteristics of the patients and DRPs. Furthermore, the normality of continuous variables was examined using the Shapiro-Wilk and Kolmogorov-Smirnov tests and by visually inspecting the histograms. None of the continuous variables had a normal distribution. Therefore, continuous variables were presented as medians (interquartile ranges, IQR). Meanwhile, logistic regression models were used to examine the potential factors associated with the totally solved status of a DRP. Multivariable analysis was adjusted for age, gender, history of thromboembolism event, history of major bleeding events, receiving services 3 times or more in 180 days, specific referral reasons, HAS-BLED score, and CHA_2_DS_2_-VASc score (only for the DOAC group). The results were presented as odds ratios (ORs) and 95% confidence intervals (95% CIs), and differences with a P value of less than 0.05 were considered statistically significant. Lastly, TTR values were compared using the Wilcoxon signed-rank test.

## Results

### Patient characteristics

This study included 112 newly referred patients, of which 78 were DOAC users, and 34 were warfarin users (Table 1). All patients were referred by physicians, 96.2% of whom were in DOAC services and 88.2% in warfarin services, from cardiovascular or cardiovascular surgery divisions. In the DOAC group, the median (IQR) age of the patients was 71.5 (66.3–78.5), 30 patients (38.5%) were female, and a median (IQR) HAS-BLED score of 2 (1–2). The most common indication was AF (96.2%, 75/78) under a median (IQR) CHA2DS2-VASc score of 3 (2–4), of which 66.7% (50/75) had paroxysmal AF. The median (IQR) service time for an initial visit was 30 (30–40) minutes, and the median (IQR) service frequency within 180 days was 2 (2–3) times. In the warfarin group, the median (IQR) age was 62.0 (56.0–68.3), younger than that of the DOAC group. In addition, it had 15 female patients (44.1%), at a slightly higher proportion than the DOAC group, and a median (IQR) HAS-BLED score of 2 (1–3). The most common indications of warfarin were mechanical valve replacement (70.6%) and AF (17.6%) under a median (IQR) CHA2DS2-VASc score of 2 (1–3), where 33.3% had paroxysmal AF. Among the 6 patients with AF taking warfarin, 4 were not under the NHI coverage for DOACs, 1 had severe mitral stenosis, and 1 was under hemodialysis. The median (IQR) service time for an initial visit was 40 (30–50) minutes, and the median (IQR) service frequency within 180 days was 2 (2–3) times. The limitation of DOAC indications resulted in the differences in patient characteristics between the groups. Lastly, the service time in the warfarin group was longer than in the DOAC group, but the numbers of visits in both groups were similar.

**Table 1:** Clinical characteristics of the participants^a^.

Variable	DOACs n = 78	Warfarin n = 34
**Age, years, median (IQR)**	71.5 (66.3–78.5)	62.0 (56.0–68.3)
≥ 65	63 (80.8)	13 (38.2)
< 65	15 (19.2)	21 (61.8)
**Female**	30 (38.5)	15 (44.1)
**Main indication of anticoagulants**		
Atrial fibrillation	75 (96.2)	6 (17.6)
Mechanical valve replacement	0 (0.0)	24 (70.6)
Others^b^	3 (3.8)	4 (11.8)
**Referral reason**		
**Specific reason**		
Initial use of OAC	19 (24.4)	3 (8.8)
OAC interruption before invasive procedure	16 (20.5)	0
ADE management	13 (16.7)	1 (2.9)
Medication adherence improvement	4 (5.1)	0
Lifestyle management	0	12 (35.3)
Drug interaction management	0	2 (5.9)
**Nonspecific reason**		
OAC counseling	16 (20.5)	11 (32.4)
OAC follow-up	10 (12.8)	5 (14.7)
**Service characteristics**		
Service time, minutes, median (IQR)	30 (30–40)	40 (30–50)
No. of visits in 180 days, median (IQR)	2 (2–3)	2 (2–3)
**CHA**_**2**_ **DS**_**2**_ **-VASc score, median (IQR)** ^c^	3 (2–4)	2 (1–3)
**HAS-BLED score, median (IQR)**	2 (1–2)	2 (1–3)
**Comorbidities**		
Hypertension	50 (64.1)	18 (52.9)
Diabetes mellitus	22 (28.2)	2 (5.9)
Coronary artery disease	17 (21.8)	5 (14.7)
Congestive heart failure	11 (14.1)	6 (17.6)
Thyroid dysfunction	5 (6.4)	0 (0.0)
Peripheral arterial disease	1 (1.3)	0 (0.0)
Active cancer	2 (2.6)	0 (0.0)
Prior cancer	12 (15.4)	4 (11.8)
Prior thromboembolism event	11 (14.1)	3 (8.8)
Stroke	6 (7.7)	2 (5.9)
Transient ischemic attack	1 (1.3)	0 (0.0)
Prior major bleeding	6 (7.7)	5 (14.7)
ALT/AST > 3 times ULN	1 (1.3)	1 (2.9)
**Co-medications**		
Rhythm control^d^	36 (46.2)	3 (8.8)
Beta-blockers^e^	32 (41.0)	16 (47.0)
Digoxin	3 (3.8)	0 (0.0)
Proton pump inhibitors	4 (5.1)	6 (17.6)
NSAIDs	9 (11.5)	2 (5.9)
**Tobacco use**	25 (32.1)	10 (29.4)
Quitted > 2 years	18 (23.1)	5 (14.7)
Quitted ≤ 2 years	0 (0.0)	2 (5.9)
Current	7 (9.0)	3 (8.8)

ADE, adverse drug event; ALT, alanine aminotransferase; AST, aspartate aminotransferase; CrCl, creatinine clearance; DOAC, direct oral anticoagulant; IQR, interquartile range; NSAIDs, non-steroid anti-inflammatory drugs; OAC, oral anticoagulant; ULN, upper limited normal.

^a^Data are presented as number (%) patients, unless otherwise noted.

^b^Warfarin group: 2 for valvular disease, 1 for deep vein thrombosis, and 1 for superior mesenteric vein thrombosis. DOACs group: 1 for deep vein thrombosis, 1 for atrial flutter, and 1 for left ventricular apical thrombus.

^c^Calculated only for participants with atrial fibrillation.

^d^Flecainide, propafenone, amiodarone, and dronedarone were included.

^e^Atenolol, bisoprolol, carvedilol, metoprolol, propranolol were included.

### DRP analysis

**Fig 1:**
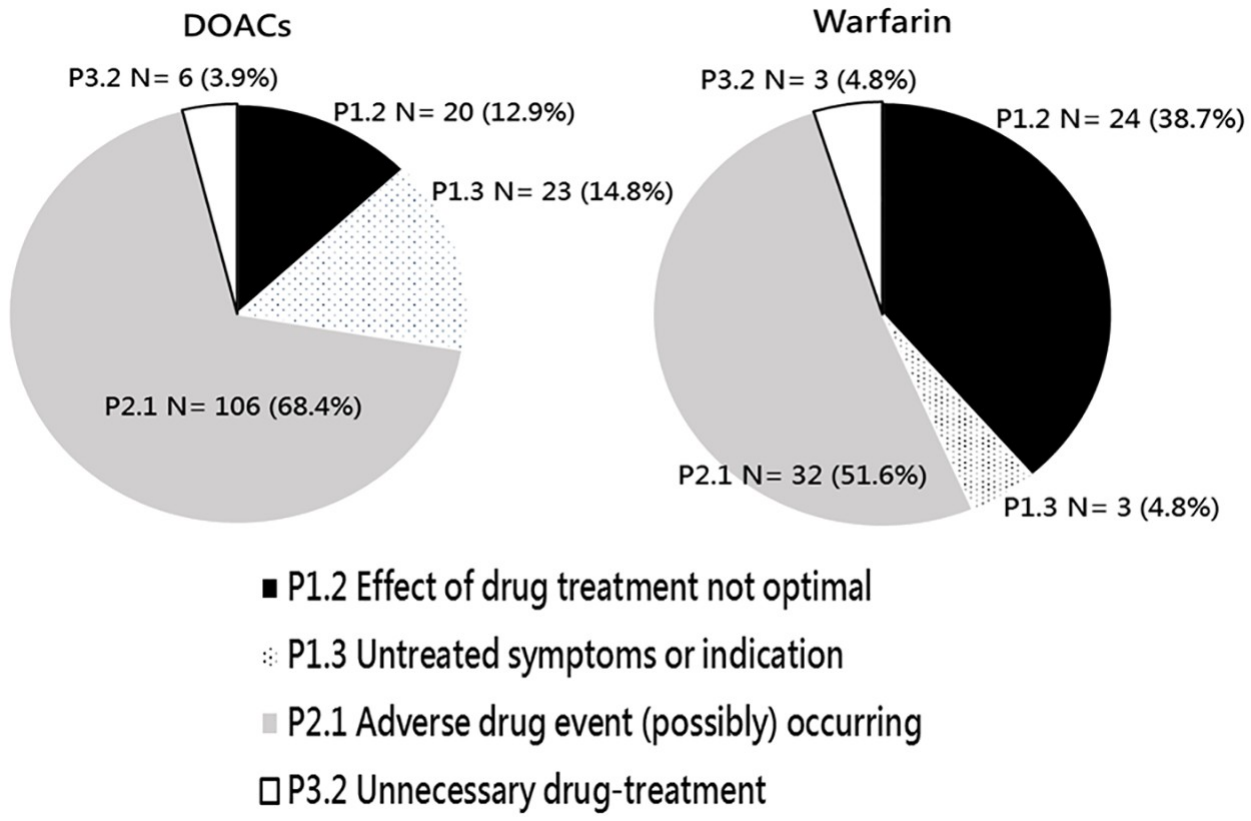
Identified DRPs according to the PCNE-DRP classification tool version 9.0. There were 155 DRPs in 78 DOAC users and 62 DRPs in 34 warfarin users. DOACs, direct oral anticoagulants; DRP, drug-related problem; PCNE, Pharmaceutical Care Network Europe.

**Fig 2:**
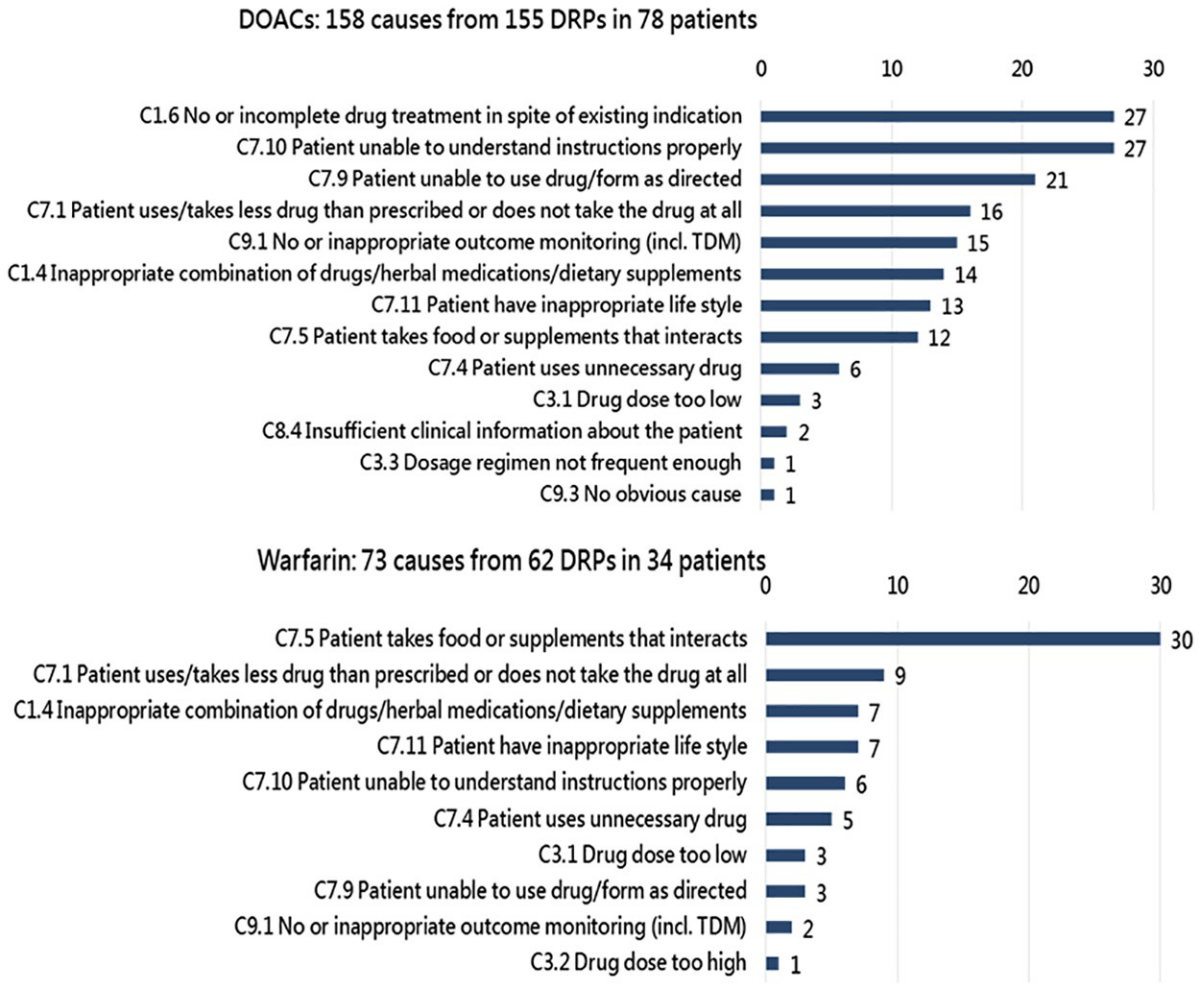
Identified causes of DRPs according to the PCNE-DRP classification tool version 9.0. DOACs, direct oral anticoagulants; DRP, drug-related problem; PCNE, Pharmaceutical Care Network Europe; TDM, therapeutic drug monitoring.

The identified DRP items in the initial service and their corresponding PCNE code are shown in the supporting information S1 and S2 Tables. In the DOAC group, 155 DRPs were identified, with an average of 2.0 DRPs per patient. The top two types of DRP were P2.1 [adverse drug event (ADE possibly) occurring] (68.4%, 106/155) and P1.3 (untreated symptoms or indication) (14.8%, 23/155) (Fig 1). Particularly, 158 causes of the identified DRPs were differentiated (Fig 2). The major causes included code C1.6 (no or incomplete drug treatment) (17.1%, 27/158), in which 25 causes were DOACs interruption for invasive procedures, and code C7.10 (patient unable to understand instructions properly for ADE management) (17.1%, 27/158), followed by code C7.9 (patient unable to use drug/form as directed) (13.3%, 21/158), in which all were initial use, and code C7.1 (patient uses less drug than prescribed or does not take the drug at all) (10.1%, 16/158), in which all were medication nonadherence). Among all causes, only 2.5% (4/158) were inappropriate dosages.

Furthermore, there were 197 interventions deployed for these DRP causes; among them, 12.2% (24/197) were at the prescriber level, 78.7% (155/197) at the patient level, and 9.1% (18/197) at the drug level (Table 2). The rate of intervention acceptance was 96.4% (190/197), the rate of fully implemented interventions were 55.8% (110/197). The rate of totally solved status for 155 DRPs was 56.8% (88/155).

**Table 2:** Interventions, acceptance, and outcome status of the identified DRPs.

Interventions	DOACs		Warfarin	
	n = 197	(100%)	n = 80	(100%)
**At prescriber level**	**24**	**(12.2)**	**7**	**(8.8)**
I1.1 Prescriber informed only	4		0	
I1.2 Prescriber asked for information	8		0	
I1.3 Intervention proposed to prescribers	4		6	
I1.4 Intervention discussed with prescribers	8		1	
**At patient level**	**155**	**(78.7)**	**68**	**(85.0)**
I2.1 Patient (drug) counselling	139		68	
I2.2 Written information provided	16		0	
**At drug level**	**18**	**(9.1)**	**5**	**(6.3)**
I3.1 Drug changed to …	1		2	
I3.2 Dosage changed to …	0		3	
I3.4 Instructions for use changed to …	15		0	
I3.5 Drug paused or stopped	2		0	
**Acceptance of the Interventions**				
**Accepted**	**190**	**(96.4)**	**79**	**(98.8)**
A1.1 Accepted and fully implemented	110		53	
A1.2 Accepted, partially implemented	26		19	
A1.3 Accepted but not implemented	29		6	
A1.4 Accepted, implementation unknown	25		1	
**Not accepted**	**6**	**(3.0)**	**1**	**(1.3)**
A2.3 Not accepted: other reason (specify)	5		1	
A2.4 Not accepted: unknown reason	1		0	
**Other** A3.1 Acceptance unknown	**1**	**(0.5)**	**0**	**(0)**
**Outcome status of the DRP**	**n = 155**	**(100%)**	**n = 62**	**(100%)**
**Solved** O1.1 Problem totally solved	**88**	**(56.8)**	**32**	**(51.6)**
**Partially solved** O2.1 Problem partially solved	**23**	**(14.8)**	**25**	**(40.3)**
**Not solved**	**30**	**(19.4)**	**4**	**(6.5)**
O3.1 Lack of cooperation of patient	14		4	
O3.3 Intervention not effective	3		0	
O3.4 No need or possibility to solve problem	13		0	
**Not known** O0.1 Problem status unknown	**14**	**(9.0)**	**1**	**(1.6)**

DOACs, direct oral anticoagulants; DRP, drug-related problem.

There were 62 DRPs identified in the warfarin group, with an average of 1.8 DRPs per patient. The two predominant types of DRP were P2.1 [ADE (possibly) occurring] (51.6%, 32/62) and P1.2 (effect of drug treatment not optimal (38.7%, 24/62) (Fig 1). For the identified DRPs, 73 causes were differentiated. The major causes were code C7.5 (patient takes food or supplements that interact) (41.1%, 30/73), in which all were drug-food/supplement interaction, followed by code C7.1 (patient uses less drug than prescribed or does not take the drug at all) (12.3%, 9/73), in which all were medication nonadherence), code C1.4 (inappropriate combination of drugs/herbal medications/dietary supplements) (9.6%, 7/73), in which all were drug-drug interaction prescribed by physicians, and code C7.11 (patient has inappropriate lifestyle) (9.6%, 7/73), in which 3 were tobacco use, 3 were alcohol abuse, and 1 was both) (Fig 2). A total of 80 interventions were deployed for these DRP causes, among which 7 were at the prescriber level, 68 at the patient level, and 5 at the drug level (Table 2). The rate of intervention acceptance was 98.8% (79/80), while the rate of fully implemented interventions were 66.3% (53/80). The rate of totally solved status for 62 DRPs was 51.6% (32/62).

The multivariable logistic regression analysis of the DOAC group showed that the service time of three or more in 180 days was associated with the totally solved status of DRPs (OR 3.11, 95% CI 1.30–7.44; Table 3). Meanwhile, no relevant factors were observed in the warfarin group.

**Table 3:** Logistic regression analysis of the factors associated with the totally solved status of DRPs.

	DOACs		Warfarin	
	Univariable	Multivariable^c^	Univariable	Multivariable^c^
	OR (95%CI)	OR (95%CI)	OR (95%CI)	OR (95%CI)
Age (years)^a^	1.03 (1.00–1.06)	1.02 (0.97–1.07)	1.01 (0.97–1.04)	1.00 (0.96–1.04)
Female	1.36 (0.71–2.62)	1.18 (0.56–2.51)	0.69 (0.25–1.91)	0.97 (0.31–3.05)
History of thromboembolism event	2.09 (0.76–5.71)	1.60 (0.46–5.55)	3.92 (0.74–20.65)	2.59 (0.31–19.98)
History of major bleeding event	0.61 (0.18–2.10)	0.49 (0.12–2.04)	0.74 (0.20–2.74)	0.76 (0.17–3.45)
Service ≥ 3 times in 180 days	2.80 (1.25–6.27)	3.11 (1.30–7.44)	1.13 (0.41–3.06)	0.81 (0.25–2.68)
Specific referral reasons^b^	0.76 (0.39–1.49)	0.85 (0.42–1.76)	1.27 (0.45–3.57)	1.38 (0.41–4.64)
HAS-BLED score^a^	1.18 (0.84–1.67)	0.86 (0.53–1.38)	1.39 (0.84–2.28)	1.32 (0.66–2.63)
CHA_2_DS_2_-VASc score^a^	1.27 (1.02–1.57)	1.18 (0.80–1.75)	N/A	N/A

DRPs, drug related problems; DOACs, direct oral anticoagulants; OR, odds ratio.

^a^ Every 1 year or point increase.

^b^ Specific reasons included initial use of anticoagulant, adverse drug reaction management, medication adherence improvement, bridging suggestions, drug-drug interaction management, life style management, as opposed to non-specific reasons such as anticoagulant follow-up or counselling.

^c^ All factors listed in the table were included in the multivariable analysis.

## DOACs and warfarin service outcomes

In both DOAC and warfarin groups, the episodes of thromboembolism events, major bleeding events, and bleeding-related hospitalizations 180 days before and after the first AC visit were compared (Table 4). The DOAC group had fewer episodes of these events after AC service.

**Table 4:** DOACs and warfarin service outcome.

	Before referral within 180 days		After referral within 180 days		P value
**DOACs**					
Thromboembolism events	2		0		
Major bleeding events	4		0		
Bleeding related hospitalizations	2		0		
**Warfarin**					
Thromboembolism events	1		1		
Major bleeding events	2		2		
Bleeding related hospitalizations	1		0		
Time in therapeutic range^a^	55.0	(36.4–79.6)	74.6	(65.3–88.8)	0.006
	TTR (%)	n	TTR (%)	n	
	<50	10	<50	4	
			50–70	2	
			>70	4	
	50–70	12	<50	1	
			50–70	3	
			>70	8	
	>70	8	>70	8	

DOACs, direct oral anticoagulants; TTR, time in therapeutic range.

^a^Data are presented as median (interquartile range).

Among the original 34 patients in the warfarin group, excluding 3 first-time users and 1 who refused warfarin treatment. The remaining 30 patients were analyzed. After 180 days of initial service, 29 patients had an improved or noninferior TTR range, indicated that the AC service was of good quality. In addition, the patients after AC visits presented a higher TTR% [median (IQR): 74.6% (65.3–88.8%)] than that before visits [median (IQR): 55.0% (36.4–79.6%)] (P = 0.006).

## Discussion

In this study, we described the types, causes, interventions, solved status of the DRPs in the patients receiving DOAC and warfarin services from a pharmacist-managed AC and analyzed the outcomes of the services. The results revealed that the factor associated with the totally solved status of DRPs was the DOAC patients receiving AC services 3 times or more in 180 days.

In our study, inappropriate dosing contributed to 2.5% of all causes of DRPs in the DOAC group, lower than previous analyses of inappropriate DOAC prescription rates (17%–24%) [18,19], likely due to the deployment of a renal function alert with dose suggestion system in our ambulatory and inpatient prescribing setting, and the facilitation of self-developed Intelligent AC Service System. Upon detecting a dosage inappropriateness, the prescribing setting initiates a warning with information of dose adjustment. Meanwhile, the self-developed Intelligent AC Service System includes DOAC regimen verifications in the standard service procedure. Although there was no referral for inappropriate DOACs dosing under the two dosage check-up system, there was still a 2.5% contribution to the causes of DRPs. This finding suggests that evaluating DOAC regimens is still an important focus in pharmacy services.

DOAC interruption for invasive procedures was one of the major interventions in the DOAC group. While warfarin usage has been practiced for over five decades, the protocols for warfarin bridging and interruption before elective surgery are familiar among medical professionals. However, the pharmacokinetics and half-lives of DOACs are uniquely different from those of warfarin. Consequently, a shorter interruption period without a bridging strategy is associated with a low rate of major bleeding and arterial thromboembolism [20]. One of the major causes of DRP is when patients receive different DOAC interruption plans between prescribing physicians and those performing invasive procedures. Therefore, evidence-based consultations and individualized interruption instructions provided by an AC meet this clinical needs. Thus, the results of this study suggest the essential role of pharmacists in the management of anticoagulant therapy, which has not been highlighted in previous studies.

Furthermore, DOACs are shown to have superior or noninferior efficacy and safety compared to warfarin. Although DOAC users take fixed doses and do not require regular coagulation tests, DOACs remain high-risk medications whose clinical outcomes could be influenced by many factors. A previous DOAC management suggestion does not emphasize the frequency of a structured follow-up, considering a 1 to 6 month interval acceptable after the follow up in the first month [21]. In contrast, our findings suggested that a frequent service of three times or more within 180 days was associated with the solved status of a DRP. In our findings, other than one-time interventions for dosage inappropriateness or the lack of renal function tests before dosing, interventions for self-use of DOAC-interacting substances, ADE management, medication nonadherence, self-care when using DOACs, and inappropriate lifestyle require regular follow-ups and multiple consultations to execute the therapeutic plans and reach the goals. Thus, a rising number of AC appointments is expected and will increase the clinical need for developing anticoagulation management tools to facilitate service efficiency [22,23]. Although further studies to specify patients’ follow-up plans are necessary, our results have provided critical insights for designing the management tools.

Our study has several important strengths. First, this is the first indepth report on the DRPs of DOAC and warfarin services within an AC. Second, the association between patient/service characteristics and DRP solved status has been analyzed.

Meanwhile, several limitations must be addressed. First, the study was conducted within a short period in a hospital. Thus, the results may not apply to other clinical settings, particularly in settings different from primarily physician referral, a combination of inperson and phone services, and service charge. Second, due to the various models of AC service, our findings may not apply to all ACs. Third, as the retrospective study design, we were unable to access all residual confounders, although we adjusted for age, sex, history of thromboemblism or major bleeding events, referral reasons, HAS-BLED score, and CHA2DS2-VASc score as covariates in logistic regression models. Finally, as with all observational studies, we could only detect associations rather than causalities. However, the results of our study provide insights into the nature of pharmacy services in ACs. Moreover, these results may serve as an important foundation for future services and research.

## Conclusions

The major types and causes of DRPs were different between DOAC and warfarin services, and the corresponding rate of totally solved status of the DRPs in the services were 56.8% and 51.6%, respectively. In addition, receiving AC services three times or more in 180 days was associated with the totally solved status of DRPs among the DOAC patients. The assessments of DRPs in AC services provide insights on the types of service for the increasing population of DOAC users. Further studies are needed to determine the impact of the frequency of pharmacist-led anticoagulation clinics on clinical outcomes for DOAC patients.

## Supporting information

S1 Fig Service protocol for the pharmacist-managed anticoagulation clinic. (TIF)

S1 Table Identified DRP items in the initial service (bold text) and their corresponding PCNE-DRP type and cause in DOAC group, n = 158. (DOCX)

S2 Table Identified DRP items in the initial service (bold text) and their corresponding PCNE-DRP type and cause in warfarin group, n = 73. (DOCX)
