# Supplementary figures and images for: Drug-related problem characterization and the solved status associated factor analysis in a pharmacist-managed anticoagulation clinic
Source: PLoS One. 2022 Aug 15;17(8):e0270263. doi: 10.1371/journal.pone.0270263 (PMC9377620; doi:10.1371/journal.pone.0270263)

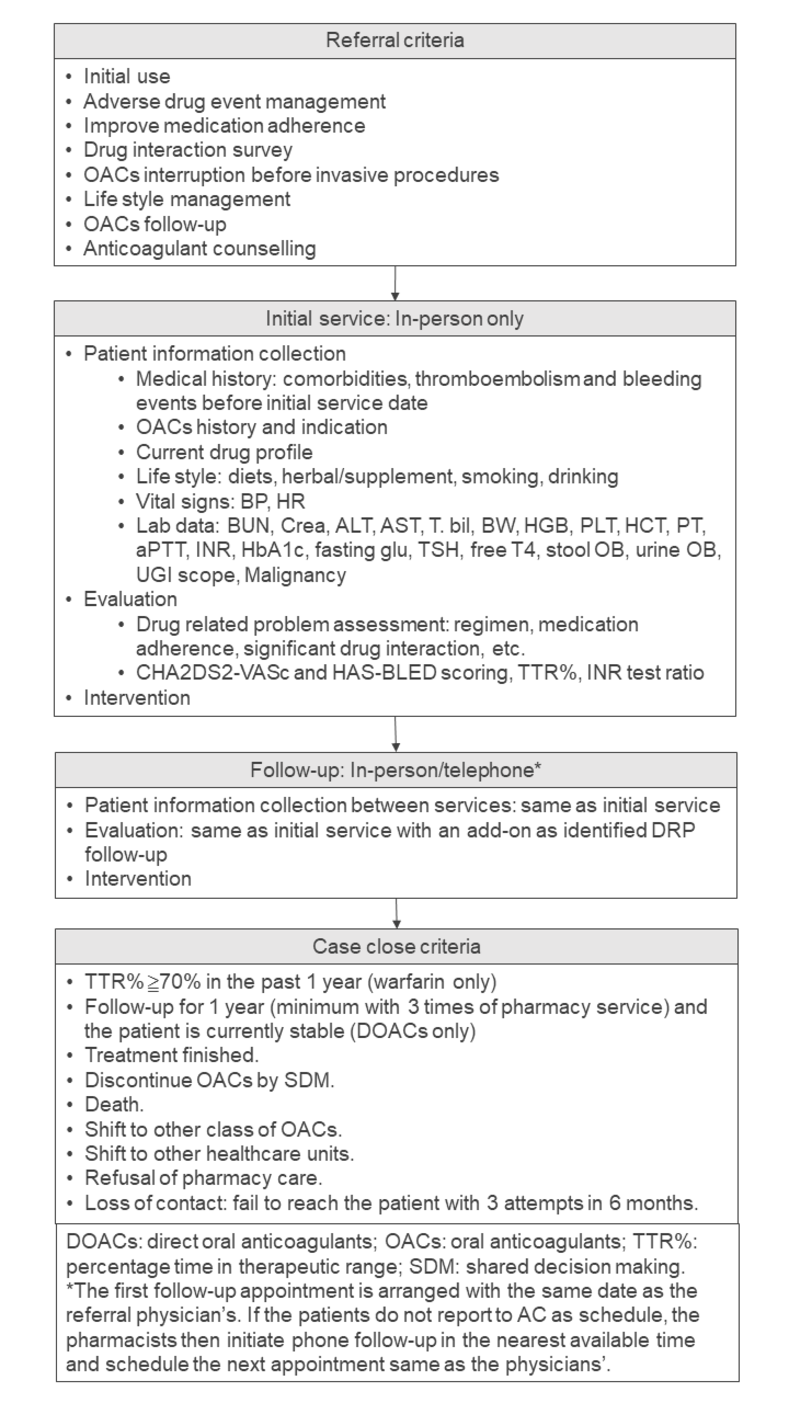

Supplement: S1 Fig — (TIF) [file pone.0270263.s001.tif]
