# Supplementary material for: Drug-related problem characterization and the solved status associated factor analysis in a pharmacist-managed anticoagulation clinic
Source: PLoS One. 2022 Aug 15;17(8):e0270263. doi: 10.1371/journal.pone.0270263 (PMC9377620; doi:10.1371/journal.pone.0270263)
Supplement: S1 Table — (DOCX) [file pone.0270263.s002.docx]

**S1 Table. Identified DRP items in the initial service (bold text) and their corresponding PCNE-DRP type and cause in DOAC group, n=158**

| Type of DRP  Cause of DRP | P1.2 Effect of drug treatment not optimal | P1.3 Untreated symptoms or indication | P2.1 Adverse drug event (possibly) occurring | P3.2 Unnecessary drug-treatment |
| --- | --- | --- | --- | --- |
| C1.4 Inappropriate combination of drugs, or drugs and herbal medications, or drugs and dietary supplements |  |  | **Drug interactions with physician prescriptions, n=14** |  |
| C1.6 No or incomplete drug treatment in spite of existing indication |  | **Untreated comorbidities or DOACs, shared decision making, n=2** | **OAC interruption before surgery, n=25** |  |
| C3.1 Drug dose too low | **Insufficient dose, n=3** |  |  |  |
| C3.3 Dosage regimen not frequent enough | **Insufficient frequency, n=1** |  |  |  |
| C7.1 Patient uses/takes less drug than prescribed or does not take the drug at all | **Compliance, n=16** |  |  |  |
| C7.10 Patient unable to understand instructions properly |  |  | **ADE self-care instruction, n=27** |  |
| C7.11 Patient have inappropriate life style |  |  | **Inappropriate life style, n=13** |  |
| C7.4 Patient uses unnecessary drug |  |  |  | **Drug interaction with self-purchased medications, n=6** |
| C7.5 Patient takes food or supplements that interacts |  |  | **Drug interaction with diet or supplements, n=12** |  |
| C7.9 Patient unable to use drug/form as directed |  | **Initial use, n=21** |  |  |
| C8.4 Insufficient clinical information about the patient |  |  | **Explanation of shifting from aspirin to DOACs, n=2** |  |
| C9.1 No or inappropriate outcome monitoring (incl. TDM) |  |  | **Renal or liver function tests, self-measured blood pressure,**  **n=15** |  |
| C9.3 No obvious cause |  |  | **n=1** |  |

*ADE* adverse drug event, *DRP* drug related problems, *DOACs* direct oral anticoagulants, OAC oral anticoagulants, *PCNE* Pharmaceutical Care Network Europe
