# Supplementary material for: Drug-related problem characterization and the solved status associated factor analysis in a pharmacist-managed anticoagulation clinic
Source: PLoS One. 2022 Aug 15;17(8):e0270263. doi: 10.1371/journal.pone.0270263 (PMC9377620; doi:10.1371/journal.pone.0270263)
Supplement: S2 Table — (DOCX) [file pone.0270263.s003.docx]

**S2 Table. Identified DRP items in the initial service (bold text) and their corresponding PCNE-DRP type and cause in warfarin group, n=73**

| Type of DRP  Cause of DRP | P1.2 Effect of drug treatment not optimal | P1.3 Untreated symptoms or indication | P2.1 Adverse drug event (possibly) occurring | P3.2 Unnecessary drug-treatment |
| --- | --- | --- | --- | --- |
| C1.4 Inappropriate combination of drugs, or drugs and herbal medications, or drugs and dietary supplements |  |  | **Drug interactions with physician prescriptions,n=7** |  |
| C3.1 Drug dose too low | **Insufficient dose, n=3** |  |  |  |
| C3.2 Drug dose too high | **Over dose, n=1** |  |  |  |
| C7.1 Patient uses/takes less drug than prescribed or does not take the drug at all | **Compliance, n=9** |  |  |  |
| C7.10 Patient unable to understand instructions properly | **Confused with dose titration, n=4** |  | **ADE self-care instruction, n=2** |  |
| C7.11 Patient have inappropriate life style | **inappropriate life style, n=2** |  | **inappropriate life style, n=5** |  |
| C7.4 Patient uses unnecessary drug | **Drug interaction with self-purchased medications, n=1** |  | **Drug interaction with self-purchased medications, n=1** | **Drug interaction with self-purchased medications, n=3** |
| C7.5 Patient takes food or supplements that interacts | **Drug interaction with diet or supplements,n=15** |  | **Drug interaction with diet or supplements, n=15** |  |
| C7.9 Patient unable to use drug/form as directed |  | **Initial use, n=3** |  |  |
| C9.1 No or inappropriate outcome monitoring (incl. TDM) |  |  | **Liver function tests, self-measured blood pressure,**  **n=2** |  |

*ADE* adverse drug event, *DRP* drug related problems, *PCNE* Pharmaceutical Care Network Europe
